# Trimethyl 4,4′,4′′-(ethene-1,1,2-tri­yl)tribenzoate

**DOI:** 10.1107/S2414314620004174

**Published:** 2020-03-31

**Authors:** Melvin J. G. Lesley, Koray Ozhan, Herman H.-Y. Sung, Ian D. Williams

**Affiliations:** a Department of Chemistry, Southern Connecticut State University, 501 Crescent, Street, New Haven, CT, 06515-1355, USA; b Department of Chemistry, The Hong Kong University of Science and Technology, Clear Water Bay, Hong Kong; Purdue University, USA

**Keywords:** crystal structure, protodeboronation, Suzuki coupling

## Abstract

The title compound, C_26_H_22_O_6_, is formed as the major product from the reaction between *syn*-1,2-bis­(pinacolatoboron)-1,2-bis­(4-methyl­carb­oxy­phen­yl)ethene and excess methyl 4-iodo­benzoate in basic DMSO using a palladium catalyst at 80°C *via* Suzuki coupling followed by protodeboronation. Crystals were grown by slow evaporation of a hexa­nes solution at room temperature.

## Abstract 2

The tandem *in situ* double Suzuki coupling reaction of a diborated ethyl­ene compound containing electron-withdrawing aromatic ester groups was studied. The major product is derived from the Suzuki coupling involving the boryl group and an aryl­iodide derivative followed by preferential protodeboronation of the second boryl ligand.

## Graphical abstract

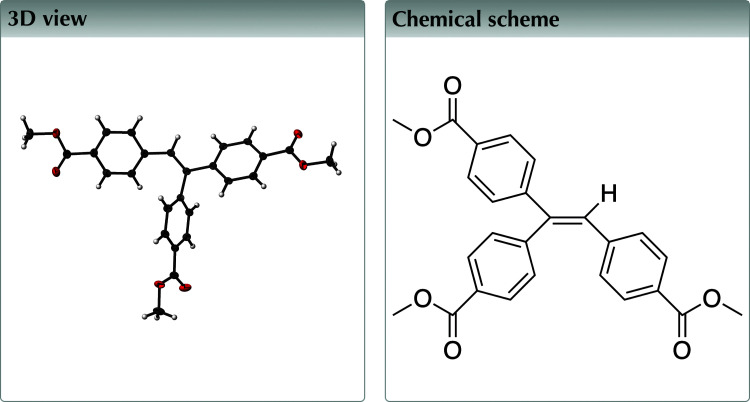

## Structure description

**Figure 1:**
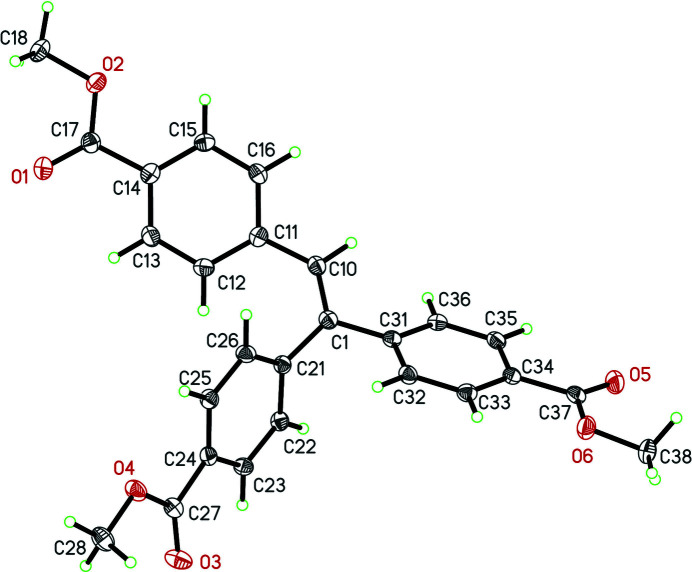
A view of the mol­ecular structure of (I). Displacement ellipsoids are drawn at the 40% probability level.

Protodeboronation is a well-known side reaction resulting in the replacement of boryl groups with hydrogen (Lee & Cheon, 2016 ▸). Initial studies of reductive deboronation have been reported for alkene (Brown & Murray, 1959 ▸, 1986 ▸) and alkyne (Brown & Zweifel, 1961 ▸; Zweifel *et al.*, 1971 ▸) derivatives under acidic conditions as an alternative method to the hydrogenation of π-bonds. More recent studies have focused on the beneficial outcomes of protodeboronation for the control of regioselectivity in reactions with aryl­boronic acid or aryl­boronate ester derivatives and heteroatomic ring structures utilizing both acidic (Beckett *et al.*, 1993 ▸; Kuivila & Nahabedian, 1961 ▸; Nahabedian & Kuivila, 1961 ▸) and basic (Lozada *et al.*, 2014 ▸) reaction conditions. Protodeboronation has also been reported for reactions involving metal catalysis employing copper (Liu *et al.*, 2014 ▸), gold (Barker *et al.*, 2015 ▸) and palladium (Lai *et al.*, 2006 ▸; Brown & Armstrong, 1996 ▸). The palladium-catalyzed Suzuki coupling reaction (Lennox & Lloyd-Jones, 2014 ▸; Suzuki, 2011 ▸; Miyaura & Suzuki, 1995 ▸) commonly employs basic conditions in hygroscopic solvents such as DMSO and DMF in addition to water for the dissolution of the base. These reactions are therefore prone to protodeboronation especially when elevated temperatures are employed. The title compound, (I), was the major product isolated in the attempted synthesis of 1,1′,2,2′-tetra­kis­(4-methyl­carb­oxy­phen­yl)ethene *via* the Pd-catalyzed double Suzuki coupling reaction (Ishiyama *et al.*, 1993 ▸; Ishiyama, Yamamoto *et al.*, 1996 ▸) between *syn*-1,2-bis­(pinacolatoboron)-1,2-bis­(4-methyl­carb­oxy­phen­yl)ethene (Ishiyama, Matsuda *et al.*, 1996 ▸) and methyl 4-iodo­benzoate. The mol­ecular structure of (I) is shown in Fig. 1 ▸.

**Figure 2:**
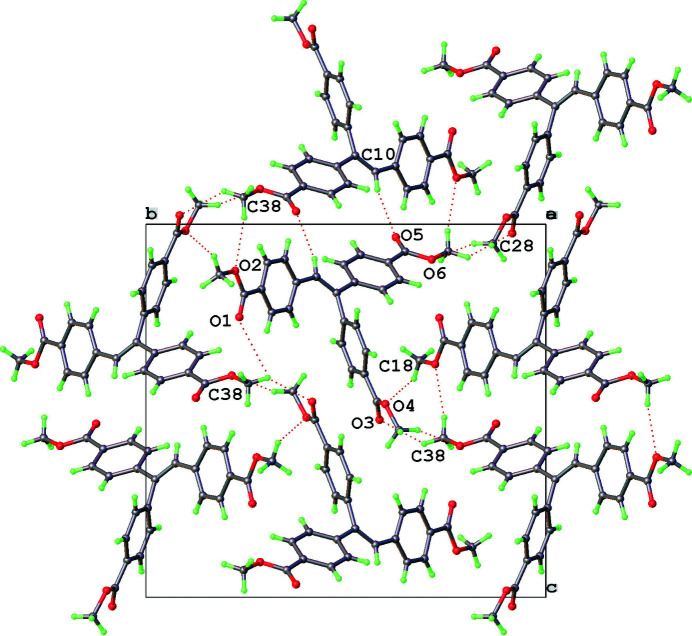
A view of the packing diagram showing short C—O⋯H—C inter­molecular inter­actions.

The title compound (I) contains four mol­ecules in the unit cell. The three methyl 4-carb­oxy­phenyl rings 1 (C11–C16), 2 (C21–C26), and 3 (C31–C36) form dihedral angles of 23.37 (6), 65.95 (4), and 33.72 (7)°, respectively, with the plane including the alkene vector (C10/C11) made up from the atoms C1, C10, C11, C21 and C31. The angles between the meth­oxy groups and the phenyl rings were calculated and indicate the groups are close to coplanar with angles of 6.3 (1)° for the mean planes defined by (C11–C16) and (C17, O2, C18); 12.5 (1)° for the mean planes defined by (C21–C26) and (C27, O4, C28); and 6.7 (2)° for the mean planes defined by (C31–C36) and (C37, O6, C38). The bond lengths and angles conform to typical value ranges (Allen *et al.*, 1987 ▸). There are a number of short C—O⋯H—C inter­molecular inter­actions (Table 1 ▸) observed in the crystal packing as shown in Fig. 2 ▸.

## Synthesis and crystallization

A 100-ml Schlenk flask was equipped with a magnetic stir bar and charged with *syn*-1,2-bis­(pinacolatoboron)-1,2-bis­(4-methyl­carb­oxy­phen­yl)ethene (3.710 g, 6.77 mmol), methyl 4-iodo­benzoate (3.723 g, 14.2 mmol), Pd_2_(dba)_3_ (0.155 g, 2.5 mol%), and *P*(*o*-tol­yl)_3_ (0.108 g, 5.25 mol%). The reaction flask was evacuated for a period of 30 minutes and placed under a dry N_2_ (*g*) atmosphere. An aqueous solution of degassed K_2_CO_3_ (2.42 ml, 7 *M*, 2.5 equiv.) was added *via* syringe followed by the addition of degassed DME (50 ml). A condenser was attached and the reaction was heated to reflux under an N_2_ atmosphere for 24 h. The reaction mixture was cooled to room temperature and water and diethyl ether were added. The orange ether layer was isolated and dried *in vacuo*. Recrystallization from ether/hexa­nes gave a white precipitate that was isolated by filtration and washed with hexane (2 × 10 ml) yielding a white solid (2.345 g, 81%; m.p. 397 K). The hexane layers were combined and slow evaporation in air gave a crop of colorless crystals of (I). Analytical data for C_26_H_22_O_6_; calculated (found): %C: 72.55 (71.28); %H: 5.15 (5.16); HRMS (EI: *m* + 1^+^) calculated (found): 431.142 (431.149); ^1^H NMR (300 MHz, CDCl_3_): 8.01 (*d*, *J* = 7.8 Hz, 2H, Ar—H), 7.99 (*d*, *J* = 7.8 Hz, 2H, Ar—H), 7.81 (*d*, *J* = 6.3 Hz, 2H, Ar—H), 7.36 (*d*, *J* = 7.8 Hz, 2H, Ar—H), 7.25 (*d*, *J* = 7.8 Hz, 2H, Ar—H), 7.12 (*s*, 1H, =CH), 7.07 (*d*, *J* = 6.3 Hz, 2H, Ar—H), 3.94 (*s*, 3H, OCH_3_), 3.93 (*s*, 3H, OCH_3_), 3.88 (*s*, 3H, OCH_3_); ^13^C{^1^H} (75 MHz, CDCl_3_): 166.71(1 C, C=O), 166.70 (1 C, C=O), 166.64 (1 C, C=O), 146.6 (1 C, **C_4_**—Ar), 144.1 (1 C, **C_4_**—Ar), 143.0 (1 C, **C_4_**—Ar), 141.0 (1 C, Ph(Ph)—**C**=), 130.4 (2 C, Ar—**C**—H), 130.1 (2 C, Ar—**C**—H), 129.8 (1 C, **C_1_**—Ar), 129.7 (overlapped 2 C, Ar—**C**—H and 1 C, =**C**H), 129.6 (1 C, **C_1_**—Ar), 129.5 (2 C, Ar—**C–**-H) 129.4 (2 C, Ar—**C**—H), 128.8 (1 C, **C_1_**—Ar), 127.6 (2 C, Ar—**C**—H), 52.22 (1 C, OCH_3_), 52.18 (1 C, OCH_3_), 52.08 (1 C, OCH_3_).

## Refinement

Crystal data, data collection and structure refinement details are summarized in Table 2 ▸.

## Supplementary Material

Crystal structure: contains datablock(s) I. DOI: 10.1107/S2414314620004174/zl4040sup1.cif

Structure factors: contains datablock(s) I. DOI: 10.1107/S2414314620004174/zl4040Isup2.hkl

CCDC reference: 1984328

Additional supporting information:  crystallographic information; 3D view; checkCIF report

## Figures and Tables

**Table 1:** Hydrogen-bond geometry (Å, °)

*D*—H⋯*A*	*D*—H	H⋯*A*	*D*⋯*A*	*D*—H⋯*A*
C10—H10⋯O5^i^	0.95	2.51	3.4082 (18)	159
C18—H18*B*⋯O4^ii^	0.98	2.72	3.5520 (19)	144
C28—H28*C*⋯O6^iii^	0.98	2.85	3.769 (2)	156
C38—H38*A*⋯O1^iv^	0.98	2.79	3.275 (2)	111
C38—H38*B*⋯O3^v^	0.98	2.57	3.339 (2)	135
C38—H38*C*⋯O2^vi^	0.98	2.66	3.595 (2)	159

Symmetry codes: (i) 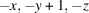 ; (ii) 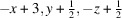 ; (iii) 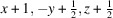 ; (iv) 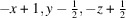 ; (v) 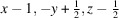 ; (vi) 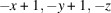 .

**Table 2:** Experimental details

Crystal data	
Chemical formula	C_26_H_22_O_6_
*M*_r_	430.43
Crystal system, space group	Monoclinic, *P*2_1_/*c*
Temperature (K)	100
*a*, *b*, *c* (Å)	6.1631 (6), 19.253 (2), 18.0743 (19)
β (°)	96.830 (1)
*V* (Å^3^)	2129.5 (4)
*Z*	4
Radiation type	Mo *K*α
μ (mm^−1^)	0.10
Crystal size (mm)	0.4 × 0.12 × 0.06

Data collection	
Diffractometer	Bruker SMART APEX CCD area detector
Absorption correction	Multi-scan (*SADABS*; Bruker, 2004 ▸)
*T*_min_, *T*_max_	0.964, 1.00
No. of measured, independent and observed [*I* > 2σ(*I*)] reflections	19788, 5132, 4358
*R*_int_	0.021
(sin θ/λ)_max_ (Å^−1^)	0.667

Refinement	
*R*[*F* ^2^ > 2σ(*F* ^2^)], *wR*(*F* ^2^), *S*	0.048, 0.125, 1.02
No. of reflections	5132
No. of parameters	292
H-atom treatment	H-atom parameters constrained
Δρ_max_, Δρ_min_ (e Å^−3^)	0.42, −0.23

Computer programs: *SMART* and *SAINT* (Bruker, 2006 ▸), *SHELXS* (Sheldrick, 2008 ▸), *SHELXL* (Sheldrick, 2015 ▸) and *OLEX2* (Dolomanov *et al.*, 2009 ▸).

## full crystallographic data

### Crystal data

**Table:**

C_26_H_22_O_6_	*F*(000) = 904
*M**_r_* = 430.43	*D*_x_ = 1.343 Mg m^−^^3^
Monoclinic, *P*2_1_/*c*	Mo *K*α radiation, λ = 0.71073 Å
*a* = 6.1631 (6) Å	Cell parameters from 6842 reflections
*b* = 19.253 (2) Å	θ = 2.3–28.2°
*c* = 18.0743 (19) Å	µ = 0.10 mm^−^^1^
β = 96.830 (1)°	*T* = 100 K
*V* = 2129.5 (4) Å^3^	Needle, colourless
*Z* = 4	0.4 × 0.12 × 0.06 mm

### Data collection

**Table:**

Bruker SMART APEX CCD area detector diffractometer	4358 reflections with *I* > 2σ(*I*)
ω and φ scans	*R*_int_ = 0.021
Absorption correction: multi-scan (SADABS; Bruker, 2004)	θ_max_ = 28.3°, θ_min_ = 1.6°
*T*_min_ = 0.964, *T*_max_ = 1.00	*h* = −8→7
19788 measured reflections	*k* = −25→25
5132 independent reflections	*l* = −24→20

### Refinement

**Table:**

Refinement on *F*^2^	Primary atom site location: structure-invariant direct methods
Least-squares matrix: full	Hydrogen site location: inferred from neighbouring sites
*R*[*F*^2^ > 2σ(*F*^2^)] = 0.048	H-atom parameters constrained
*wR*(*F*^2^) = 0.125	*w* = 1/[σ^2^(*F*_o_^2^) + (0.0603*P*)^2^ + 1.2194*P*] where *P* = (*F*_o_^2^ + 2*F*_c_^2^)/3
*S* = 1.02	(Δ/σ)_max_ < 0.001
5132 reflections	Δρ_max_ = 0.42 e Å^−^^3^
292 parameters	Δρ_min_ = −0.23 e Å^−^^3^
0 restraints	

### Special details

**Table:**

Geometry. All esds (except the esd in the dihedral angle between two l.s. planes) are estimated using the full covariance matrix. The cell esds are taken into account individually in the estimation of esds in distances, angles and torsion angles; correlations between esds in cell parameters are only used when they are defined by crystal symmetry. An approximate (isotropic) treatment of cell esds is used for estimating esds involving l.s. planes.
Refinement. Hydrogen atoms were placed geometrically and treated with riding constraints and thermal parameters derived from the C atoms to which they were attached. All –CH and CH_2_ groups had H—*U*_iso_ fixed at 1.2 times the C atom. Methyls were idealized as freely rotating CH_3_ groups with H—*U*_iso_ fixed at 1.5 times that of the C atom.

### Fractional atomic coordinates and isotropic or equivalent isotropic displacement parameters (Å^2^)

**Table:**

	*x*	*y*	*z*	*U*_iso_*/*U*_eq_	
O1	1.4240 (2)	0.76631 (7)	0.24887 (7)	0.0368 (3)	
O2	1.39707 (17)	0.77704 (6)	0.12488 (6)	0.0271 (2)	
O3	0.73744 (19)	0.41585 (6)	0.52402 (6)	0.0316 (3)	
O4	1.06230 (17)	0.40164 (6)	0.48215 (6)	0.0276 (2)	
O5	−0.37892 (18)	0.37535 (6)	0.03473 (6)	0.0307 (3)	
O6	−0.20613 (18)	0.28543 (6)	0.09445 (6)	0.0286 (3)	
C1	0.5152 (2)	0.52320 (7)	0.18403 (8)	0.0195 (3)	
C10	0.5799 (2)	0.57397 (7)	0.14079 (8)	0.0209 (3)	
H10	0.4962	0.5785	0.0934	0.025*	
C11	0.7611 (2)	0.62360 (7)	0.15587 (8)	0.0204 (3)	
C12	0.8586 (2)	0.64214 (8)	0.22724 (8)	0.0251 (3)	
H12	0.7998	0.6249	0.2699	0.030*	
C13	1.0391 (2)	0.68521 (8)	0.23623 (9)	0.0260 (3)	
H13	1.1035	0.6972	0.2849	0.031*	
C14	1.1273 (2)	0.71115 (7)	0.17448 (8)	0.0219 (3)	
C15	1.0257 (2)	0.69628 (7)	0.10334 (8)	0.0216 (3)	
H15	1.0810	0.7155	0.0609	0.026*	
C16	0.8438 (2)	0.65346 (7)	0.09441 (8)	0.0215 (3)	
H16	0.7739	0.6442	0.0457	0.026*	
C17	1.3300 (2)	0.75414 (8)	0.18812 (9)	0.0236 (3)	
C18	1.5992 (2)	0.81570 (8)	0.13491 (10)	0.0286 (3)	
H18A	1.5806	0.8571	0.1650	0.043*	
H18B	1.6390	0.8297	0.0862	0.043*	
H18C	1.7152	0.7865	0.1604	0.043*	
C21	0.6187 (2)	0.50418 (7)	0.26047 (8)	0.0189 (3)	
C22	0.4967 (2)	0.51013 (7)	0.32031 (8)	0.0201 (3)	
H22	0.3573	0.5318	0.3132	0.024*	
C23	0.5766 (2)	0.48481 (7)	0.39003 (8)	0.0204 (3)	
H23	0.4915	0.4888	0.4303	0.024*	
C24	0.7814 (2)	0.45347 (7)	0.40100 (8)	0.0189 (3)	
C25	0.9079 (2)	0.44951 (8)	0.34232 (8)	0.0212 (3)	
H25	1.0498	0.4296	0.3501	0.025*	
C26	0.8270 (2)	0.47469 (8)	0.27240 (8)	0.0220 (3)	
H26	0.9139	0.4718	0.2325	0.026*	
C27	0.8533 (2)	0.42235 (7)	0.47540 (8)	0.0216 (3)	
C28	1.1394 (3)	0.36749 (9)	0.55144 (9)	0.0318 (4)	
H28A	1.0950	0.3944	0.5932	0.048*	
H28B	1.2992	0.3642	0.5564	0.048*	
H28C	1.0765	0.3208	0.5518	0.048*	
C31	0.3270 (2)	0.47814 (7)	0.15514 (8)	0.0188 (3)	
C32	0.3224 (2)	0.40802 (8)	0.17503 (8)	0.0207 (3)	
H32	0.4409	0.3891	0.2072	0.025*	
C33	0.1488 (2)	0.36576 (7)	0.14883 (8)	0.0216 (3)	
H33	0.1491	0.3181	0.1626	0.026*	
C34	−0.0268 (2)	0.39329 (7)	0.10213 (8)	0.0200 (3)	
C35	−0.0243 (2)	0.46303 (8)	0.08209 (8)	0.0210 (3)	
H35	−0.1436	0.4820	0.0504	0.025*	
C36	0.1507 (2)	0.50481 (7)	0.10801 (8)	0.0205 (3)	
H36	0.1510	0.5523	0.0936	0.025*	
C37	−0.2229 (2)	0.35197 (8)	0.07316 (8)	0.0220 (3)	
C38	−0.3997 (3)	0.24411 (9)	0.07264 (10)	0.0312 (4)	
H38A	−0.5224	0.2627	0.0964	0.047*	
H38B	−0.3724	0.1959	0.0884	0.047*	
H38C	−0.4353	0.2458	0.0184	0.047*	

### Atomic displacement parameters (Å^2^)

**Table:**

	*U*^11^	*U*^22^	*U*^33^	*U*^12^	*U*^13^	*U*^23^
O1	0.0355 (6)	0.0451 (7)	0.0285 (6)	−0.0181 (5)	−0.0013 (5)	−0.0022 (5)
O2	0.0243 (5)	0.0288 (6)	0.0287 (6)	−0.0083 (4)	0.0056 (4)	−0.0029 (4)
O3	0.0357 (6)	0.0374 (6)	0.0231 (6)	0.0127 (5)	0.0088 (5)	0.0084 (5)
O4	0.0238 (5)	0.0371 (6)	0.0208 (5)	0.0077 (4)	−0.0018 (4)	0.0055 (4)
O5	0.0261 (6)	0.0356 (6)	0.0282 (6)	−0.0044 (5)	−0.0060 (5)	0.0026 (5)
O6	0.0270 (5)	0.0254 (5)	0.0320 (6)	−0.0065 (4)	−0.0029 (4)	−0.0014 (4)
C1	0.0184 (6)	0.0222 (7)	0.0178 (7)	0.0020 (5)	0.0010 (5)	−0.0010 (5)
C10	0.0200 (6)	0.0237 (7)	0.0184 (7)	0.0007 (5)	0.0000 (5)	−0.0005 (5)
C11	0.0176 (6)	0.0192 (7)	0.0241 (7)	0.0010 (5)	0.0015 (5)	0.0003 (5)
C12	0.0288 (7)	0.0271 (7)	0.0197 (7)	−0.0048 (6)	0.0042 (6)	0.0022 (6)
C13	0.0277 (7)	0.0282 (8)	0.0208 (7)	−0.0037 (6)	−0.0022 (6)	−0.0006 (6)
C14	0.0203 (6)	0.0190 (6)	0.0265 (7)	−0.0008 (5)	0.0026 (5)	−0.0009 (5)
C15	0.0229 (7)	0.0206 (7)	0.0220 (7)	0.0003 (5)	0.0050 (5)	0.0021 (5)
C16	0.0235 (7)	0.0211 (7)	0.0192 (7)	0.0016 (5)	−0.0011 (5)	−0.0009 (5)
C17	0.0241 (7)	0.0210 (7)	0.0254 (7)	−0.0014 (5)	0.0014 (6)	−0.0018 (6)
C18	0.0229 (7)	0.0262 (8)	0.0373 (9)	−0.0079 (6)	0.0067 (6)	−0.0047 (6)
C21	0.0191 (6)	0.0191 (6)	0.0180 (6)	−0.0007 (5)	0.0006 (5)	0.0005 (5)
C22	0.0171 (6)	0.0222 (7)	0.0206 (7)	0.0028 (5)	0.0007 (5)	0.0004 (5)
C23	0.0209 (6)	0.0223 (7)	0.0187 (7)	0.0017 (5)	0.0050 (5)	−0.0003 (5)
C24	0.0200 (6)	0.0184 (6)	0.0176 (6)	0.0005 (5)	−0.0002 (5)	−0.0001 (5)
C25	0.0166 (6)	0.0258 (7)	0.0206 (7)	0.0033 (5)	0.0006 (5)	0.0005 (5)
C26	0.0199 (6)	0.0282 (7)	0.0187 (7)	0.0026 (5)	0.0051 (5)	0.0009 (6)
C27	0.0248 (7)	0.0203 (7)	0.0192 (7)	0.0038 (5)	0.0004 (5)	0.0000 (5)
C28	0.0354 (8)	0.0336 (8)	0.0243 (8)	0.0096 (7)	−0.0056 (6)	0.0061 (6)
C31	0.0180 (6)	0.0218 (7)	0.0168 (6)	−0.0008 (5)	0.0035 (5)	−0.0014 (5)
C32	0.0192 (6)	0.0233 (7)	0.0190 (7)	0.0028 (5)	−0.0010 (5)	0.0023 (5)
C33	0.0255 (7)	0.0187 (6)	0.0207 (7)	−0.0003 (5)	0.0034 (5)	0.0010 (5)
C34	0.0199 (6)	0.0247 (7)	0.0155 (6)	−0.0028 (5)	0.0032 (5)	−0.0031 (5)
C35	0.0197 (6)	0.0270 (7)	0.0160 (6)	0.0022 (5)	0.0004 (5)	0.0028 (5)
C36	0.0226 (7)	0.0201 (6)	0.0189 (7)	0.0013 (5)	0.0028 (5)	0.0037 (5)
C37	0.0241 (7)	0.0259 (7)	0.0163 (7)	−0.0018 (6)	0.0038 (5)	−0.0025 (5)
C38	0.0295 (8)	0.0297 (8)	0.0331 (9)	−0.0109 (6)	−0.0017 (7)	−0.0055 (7)

### Geometric parameters (Å, º)

**Table:**

O1—C17	1.2021 (19)	C21—C22	1.3934 (19)
O2—C17	1.3356 (19)	C21—C26	1.3967 (19)
O2—C18	1.4439 (17)	C22—H22	0.9500
O3—C27	1.2030 (18)	C22—C23	1.3857 (19)
O4—C27	1.3400 (17)	C23—H23	0.9500
O4—C28	1.4439 (18)	C23—C24	1.3921 (19)
O5—C37	1.2046 (18)	C24—C25	1.391 (2)
O6—C37	1.3380 (19)	C24—C27	1.4905 (19)
O6—C38	1.4491 (17)	C25—H25	0.9500
C1—C10	1.341 (2)	C25—C26	1.390 (2)
C1—C21	1.4968 (19)	C26—H26	0.9500
C1—C31	1.4922 (19)	C28—H28A	0.9800
C10—H10	0.9500	C28—H28B	0.9800
C10—C11	1.4704 (19)	C28—H28C	0.9800
C11—C12	1.403 (2)	C31—C32	1.398 (2)
C11—C16	1.399 (2)	C31—C36	1.3963 (19)
C12—H12	0.9500	C32—H32	0.9500
C12—C13	1.382 (2)	C32—C33	1.382 (2)
C13—H13	0.9500	C33—H33	0.9500
C13—C14	1.391 (2)	C33—C34	1.396 (2)
C14—C15	1.391 (2)	C34—C35	1.391 (2)
C14—C17	1.495 (2)	C34—C37	1.4891 (19)
C15—H15	0.9500	C35—H35	0.9500
C15—C16	1.386 (2)	C35—C36	1.382 (2)
C16—H16	0.9500	C36—H36	0.9500
C18—H18A	0.9800	C38—H38A	0.9800
C18—H18B	0.9800	C38—H38B	0.9800
C18—H18C	0.9800	C38—H38C	0.9800
			
C17—O2—C18	114.39 (12)	C23—C24—C27	118.03 (12)
C27—O4—C28	115.36 (12)	C25—C24—C23	119.75 (13)
C37—O6—C38	114.49 (12)	C25—C24—C27	122.17 (12)
C10—C1—C21	126.32 (13)	C24—C25—H25	120.0
C10—C1—C31	119.49 (13)	C26—C25—C24	120.08 (13)
C31—C1—C21	114.16 (12)	C26—C25—H25	120.0
C1—C10—H10	115.2	C21—C26—H26	119.8
C1—C10—C11	129.60 (13)	C25—C26—C21	120.40 (13)
C11—C10—H10	115.2	C25—C26—H26	119.8
C12—C11—C10	124.65 (13)	O3—C27—O4	123.37 (13)
C16—C11—C10	117.39 (13)	O3—C27—C24	124.22 (13)
C16—C11—C12	117.95 (13)	O4—C27—C24	112.41 (12)
C11—C12—H12	119.6	O4—C28—H28A	109.5
C13—C12—C11	120.77 (14)	O4—C28—H28B	109.5
C13—C12—H12	119.6	O4—C28—H28C	109.5
C12—C13—H13	119.8	H28A—C28—H28B	109.5
C12—C13—C14	120.47 (14)	H28A—C28—H28C	109.5
C14—C13—H13	119.8	H28B—C28—H28C	109.5
C13—C14—C15	119.48 (13)	C32—C31—C1	120.65 (12)
C13—C14—C17	117.72 (13)	C36—C31—C1	121.06 (13)
C15—C14—C17	122.80 (13)	C36—C31—C32	118.29 (12)
C14—C15—H15	120.0	C31—C32—H32	119.4
C16—C15—C14	119.93 (13)	C33—C32—C31	121.24 (13)
C16—C15—H15	120.0	C33—C32—H32	119.4
C11—C16—H16	119.4	C32—C33—H33	120.1
C15—C16—C11	121.20 (13)	C32—C33—C34	119.78 (13)
C15—C16—H16	119.4	C34—C33—H33	120.1
O1—C17—O2	123.56 (14)	C33—C34—C37	123.26 (13)
O1—C17—C14	124.15 (14)	C35—C34—C33	119.49 (13)
O2—C17—C14	112.30 (12)	C35—C34—C37	117.24 (13)
O2—C18—H18A	109.5	C34—C35—H35	119.8
O2—C18—H18B	109.5	C36—C35—C34	120.39 (13)
O2—C18—H18C	109.5	C36—C35—H35	119.8
H18A—C18—H18B	109.5	C31—C36—H36	119.6
H18A—C18—H18C	109.5	C35—C36—C31	120.81 (13)
H18B—C18—H18C	109.5	C35—C36—H36	119.6
C22—C21—C1	119.09 (12)	O5—C37—O6	123.55 (13)
C22—C21—C26	118.98 (13)	O5—C37—C34	124.19 (14)
C26—C21—C1	121.67 (12)	O6—C37—C34	112.26 (12)
C21—C22—H22	119.6	O6—C38—H38A	109.5
C23—C22—C21	120.73 (13)	O6—C38—H38B	109.5
C23—C22—H22	119.6	O6—C38—H38C	109.5
C22—C23—H23	120.0	H38A—C38—H38B	109.5
C22—C23—C24	119.99 (13)	H38A—C38—H38C	109.5
C24—C23—H23	120.0	H38B—C38—H38C	109.5

### Hydrogen-bond geometry (Å, º)

**Table:**

*D*—H···*A*	*D*—H	H···*A*	*D*···*A*	*D*—H···*A*
C10—H10···O5^i^	0.95	2.51	3.4082 (18)	159
C18—H18*B*···O4^ii^	0.98	2.72	3.5520 (19)	144
C28—H28*C*···O6^iii^	0.98	2.85	3.769 (2)	156
C38—H38*A*···O1^iv^	0.98	2.79	3.275 (2)	111
C38—H38*B*···O3^v^	0.98	2.57	3.339 (2)	135
C38—H38*C*···O2^vi^	0.98	2.66	3.595 (2)	159

Symmetry codes: (i) −*x*, −*y*+1, −*z*; (ii) −*x*+3, *y*+1/2, −*z*+1/2; (iii) *x*+1, −*y*+1/2, *z*+1/2; (iv) −*x*+1, *y*−1/2, −*z*+1/2; (v) *x*−1, −*y*+1/2, *z*−1/2; (vi) −*x*+1, −*y*+1, −*z*.
